# The mechanism of action and experimental verification of Gan-song Yin on renal clear cell carcinoma based on network pharmacology and bioinformatics

**DOI:** 10.1007/s12672-024-00909-1

**Published:** 2024-02-28

**Authors:** Wenjie Jiang, Ling Yuan, Qian Liu, Xiangyang Li, Yifan Yang, Jiaqing Li, Taiqiang Jiao, Yang Niu, Lei Zhang, Hongli Dou, Yi Nan

**Affiliations:** 1 Key Laboratory of Ningxia Minority Medicine Modernization Ministry of Education, Ningxia Medical University, Yinchuan, 750004 Ningxia China; 2 School of Traditional Chinese Medicine, Ningxia Medical University, Yinchuan, 750004 Ningxia China; 3 Shaanxi University of Traditional Chinese Medicine, Xianyang, 712046 Shaanxi China; 4 College of Pharmacy, Ningxia Medical University, Yinchuan, 750004 Ningxia China; 5 School of Clinical Medicine, Ningxia Medical University, Yinchuan, 750004 Ningxia China

**Keywords:** Gan-song Yin, Renal clear cell carcinoma, Network pharmacology, Bioinformatics, Experimental verification

## Abstract

**Background:**

Gan-song Yin (GSY) is originated from the scripture “Gan-song Pills”, a medical work of the Ningxia ethnic minorities, and its treatment of kidney diseases has good results. Its method of treating Renal clear cell carcinoma (KIRC) is still unknown, nevertheless.

**Methods:**

Firstly, utilizing a network pharmacology strategy to screen GSY for active components and targets and looking up KIRC-related targets in GeneCards and GEO databases. Secondly, protein interaction networks were constructed and analyzed for GO and KEGG enrichment. Molecular docking was then performed and clinical and other correlations of the network pharmacology results were analyzed using bioinformatic analysis methods. Finally, we performed in vitro cellular experiments with 786-O cells and ACHN cells to validate the results of network pharmacology and bioinformatic analysis.

**Results:**

With the help of network pharmacological analysis, six hub targets were eliminated. Bioinformatics study revealed that the hub targets has clinically significant clinical guiding importance. The results showed that GSY inhibited the proliferation of 786-O cells and ACHN cells, induced cell apoptosis, blocked cell cycle, and reduced cell colony formation ability. qRT-PCR results showed that GSY promoted the expression of *ALB* and *CASP3* genes, and inhibited the expression of *EGFR*, *JUN*, *MYC* and *VEGFA* genes. Western blot results showed that GSY could promote the expression of ALB and CASP3 protein, and inhibit the expression of EGFR, JUN, MYC and VEGFA protein.

**Conclusions:**

Network pharmacology and bioinformatics analysis showed that GSY could act on multiple targets through a variety of components to achieve the effect of treating KIRC. In this study, we confirmed that GSY inhibits KIRC by regulating the expression of core targets through in vitro cellular experiments, thus providing a reference for subsequent related studies.

## Introduction

Renal carcinoma (RCC) is a malignant tumor originating from the renal parenchyma urothelial system, with increasing incidence, especially in the young population [1, 2]. In 2020, there were more than 431,000 new cases and 179,000 deaths from kidney cancer worldwide [3]. Renal clear cell carcinoma (KIRC) is the most common pathological subtype of renal cancer. Since it is not sensitive to radiotherapy and chemotherapy, it is of great significance to search for new natural drugs with high efficiency, low cost and good tolerance for KIRC treatment. Due to its “multi-component—multi-target—multi-pathway—multi-link” mode of action, Traditional Chinese Medicine (TCM) has shown great potential in controlling disease symptoms, delaying the process and promoting prognosis, etc. Therefore, studying the mechanism of action of TCM in treating KIRC is undoubtedly a beneficial attempt to explore new ideas, new methods and new drugs on the road of KIRC prevention and treatment.

In recent years, more and more pharmacologists and chemists have explored the anti-tumor treatment of TCM components in China, providing new ideas for modern anti-tumor research. The experiment of Liao Hongliang [4] et al. found that the Clerodendranthus spicatus (Thunb) C.Y.Wu can induce the apoptosis of KIRC cells through MAPK and p53 pathways. Soft Kin Dispersing Formula (From Shanxi Hospital of Traditional Chinese Medicine, it is composed of cat’s claw grass, mountain mushroom, Zedoary, Wolfberry, summer hay, Thunberg Fritillaria, gecko, chicken gold, vinegar mountain beetle and oyster). suppresses the implantation tumor growth in nude mice with 786-O cells, and its mechanism of action is related to the targeted blockade of Notch signaling expression, which further induces apoptosis [5]. The herbal extract ZPT inhibited the proliferation of 786-O cells and 769-P cells, and also inhibited the clone formation rate of 786-O and 769-P, and increased the apoptosis of KIRC cells [6]. Aestin can effectively inhibit the proliferation of 786-O cells and SN12-PM6 cells, block cell cycle and induce cell apoptosis [7]. Liao Can [8] et al. found that chloroform extract of Lungwort inhibited the proliferation and promoted the apoptosis of KIRC cells by inhibiting the activation of PI3K/AKT signaling pathway and down-regulating the protein expression of p-AKT, PI3K, and p-PI3K. Buyi Qushi Huoxue Recipe inhibits the proliferation of 786–0 cells and promotes its apoptosis by regulating the expression of PI3K, AKT, and NF-kB signaling pathways [9]. Shikonin inhibited the proliferation of human 786-O cells with time and dose effects [10]. It can therefore be demonstrated that Chinese medicine has been increasingly emphasized in the treatment of KIRC and has achieved certain results.

Gan-song Yin (GSY) is derived from the Ningxia minority medical book “Gan-song Pills”, which is composed of 13 traditional Chinese medicines such as nard pine, woody, cumin, clove, agarwood, matsutake mushroom, rhubarb, wolfberry, dodder, aloe, ginseng, cistanche and safflower [11]. Previous studies have shown that [12–14]: GSY can alleviate renal pathological injury and has protective effects on renal structure and function in DKD rats. In vitro cell experiments also confirmed that GSY had a positive recovery effect on the damage of renal tubular epithelial cells and podocytes induced by high glucose. In addition, hundreds of patients with type II DKD in Ningxia region benefited from its clinical application. However, its relationship with KIRC is unclear.

**Fig. 1:**
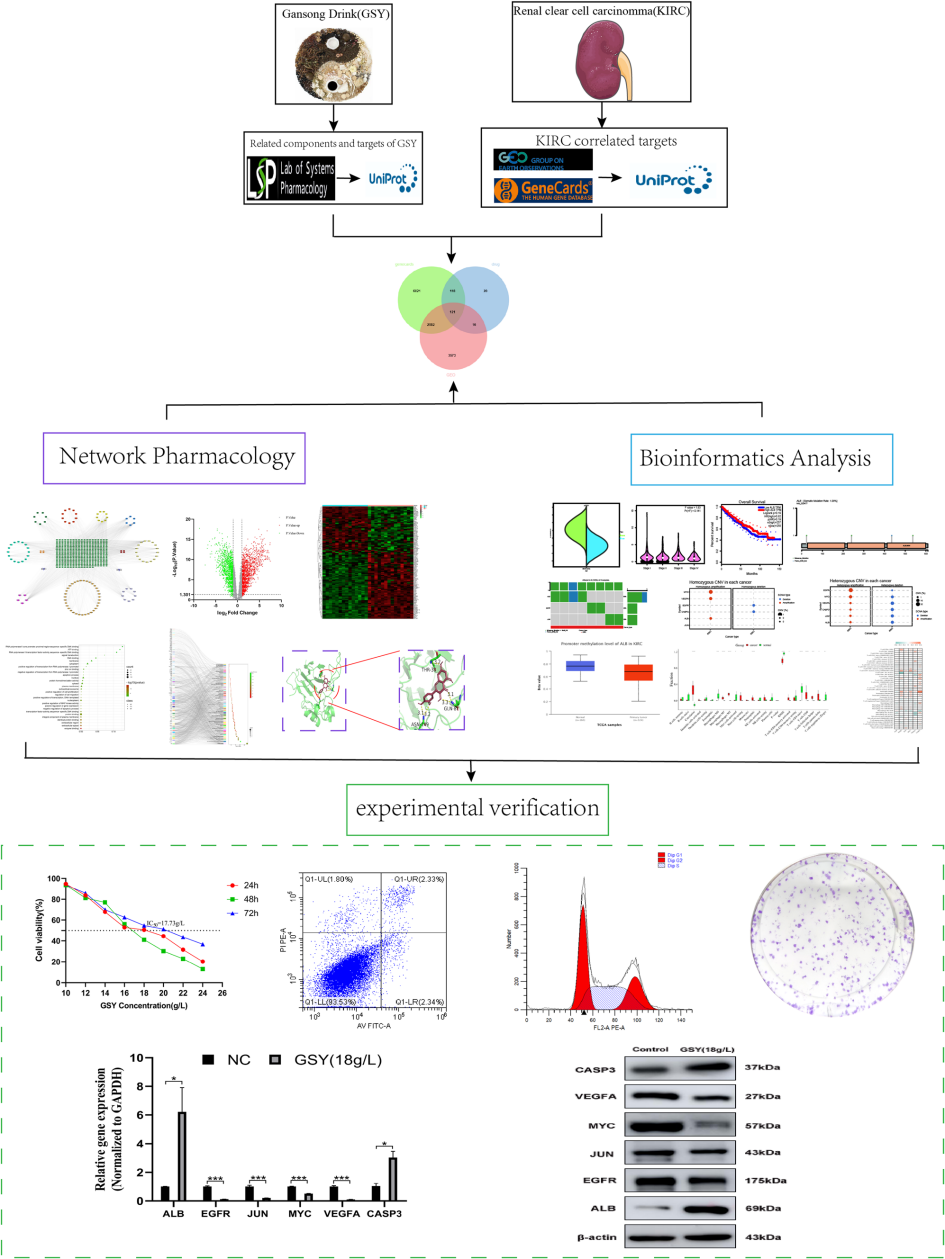
Flowchart

In recent years, network pharmacology and bioinformatics have been increasingly used in the field of TCM due to its multi-component, multi-target and multi-pathway characteristics. Based on this, we predicted the targets of GSY in the treatment of KIRC by network pharmacology, screened out the key active components such as quercetin, and the core targets such as ALB, CASP3, and JUN. Finally, experiments verify the results by kidney cells, can prove GSY through regulating the core target ALB, CASP3 and JUN expression, inhibition of 786–O cells and ACHN cells proliferation, promote cell apoptosis and block cycle, reduce the cell clone formation ability. Therefore, this study employed a combination of network pharmacology, bioinformatics analysis, and experimental verification to validate the efficacy of GSY in treating KIRC, aiming to provide valuable insights for future research on GSY's therapeutic potential in KIRC treatment. The specific process is shown in Fig. 1.

## Results

### Results of network pharmacology and bioinformatics analysis

#### Prediction of GSY and KIRC targets

**Fig. 2:**
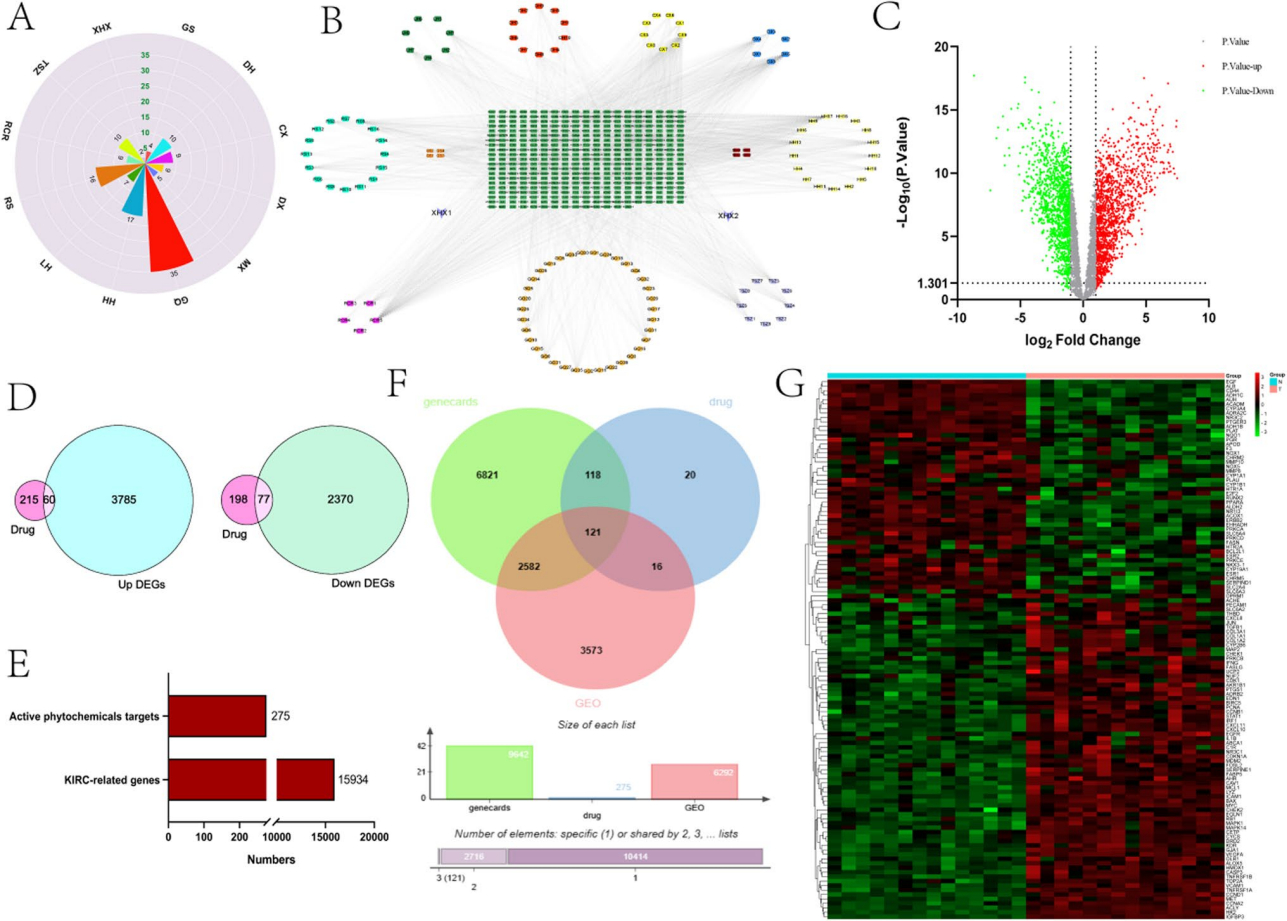
Prediction of GSY and KIRC targets. (**A**) Number of active ingredients in the drug composition of GSY (B) PPI network of GSY active ingredients and targets, with green in the center representing the target and the surrounding part representing the active ingredient (**C**) Volcano plot of differentially expressed genes in KIRC. Red represents up-regulated genes, green down-regulated genes, and gray represents genes that are undifferentially expressed (**D**) Venn diagram of intersection targets of GSY and KIRC up-regulation and down-regulation (**E**) Entry plot of GSY and KIRC targets (**F**) Venn’s plot of intersecting targets of GSY and KIRC (**G**) Heat maps of 121 intersection targets, with red indicating high gene expression and green indicating low gene expression

The total number of TCM ingredients of GSY obtained from TCMSP database was 133 (Fig. 2A). There are 275 GSY targets, and the PPI network diagram with components is shown in Fig. 2B. The GSE66270 data set was obtained from the GEO database, and 6292 KIRC targets were obtained after deduplication. Volcano plot analysis of 6292 KIRC targets using the Graphpad prism language yielded 3845 up-regulated genes and 2447 down-regulated genes, where green indicates down-regulated genes and red indicates up-regulated genes (Fig. 2C). 275 drug targets were intersected with 3845 disease up-regulated gene targets and 2447 down-regulated gene targets, and 60 and 77 intersection targets were obtained respectively (Fig. 2D). A total of 9642 KIRC targets were obtained from GeneCards, which were matched with 275 GSY targets and 6292 differentially expressed genes to obtain 121 common targets (Fig. 2F). The heat map shows that the expression of intersection targets in KIRC is 1/2 up and 1/2 down (Fig. 2G). The bar chart of KIRC target and GSY target is shown in Fig. 2E.

#### Determination of the hub targets

**Fig. 3:**
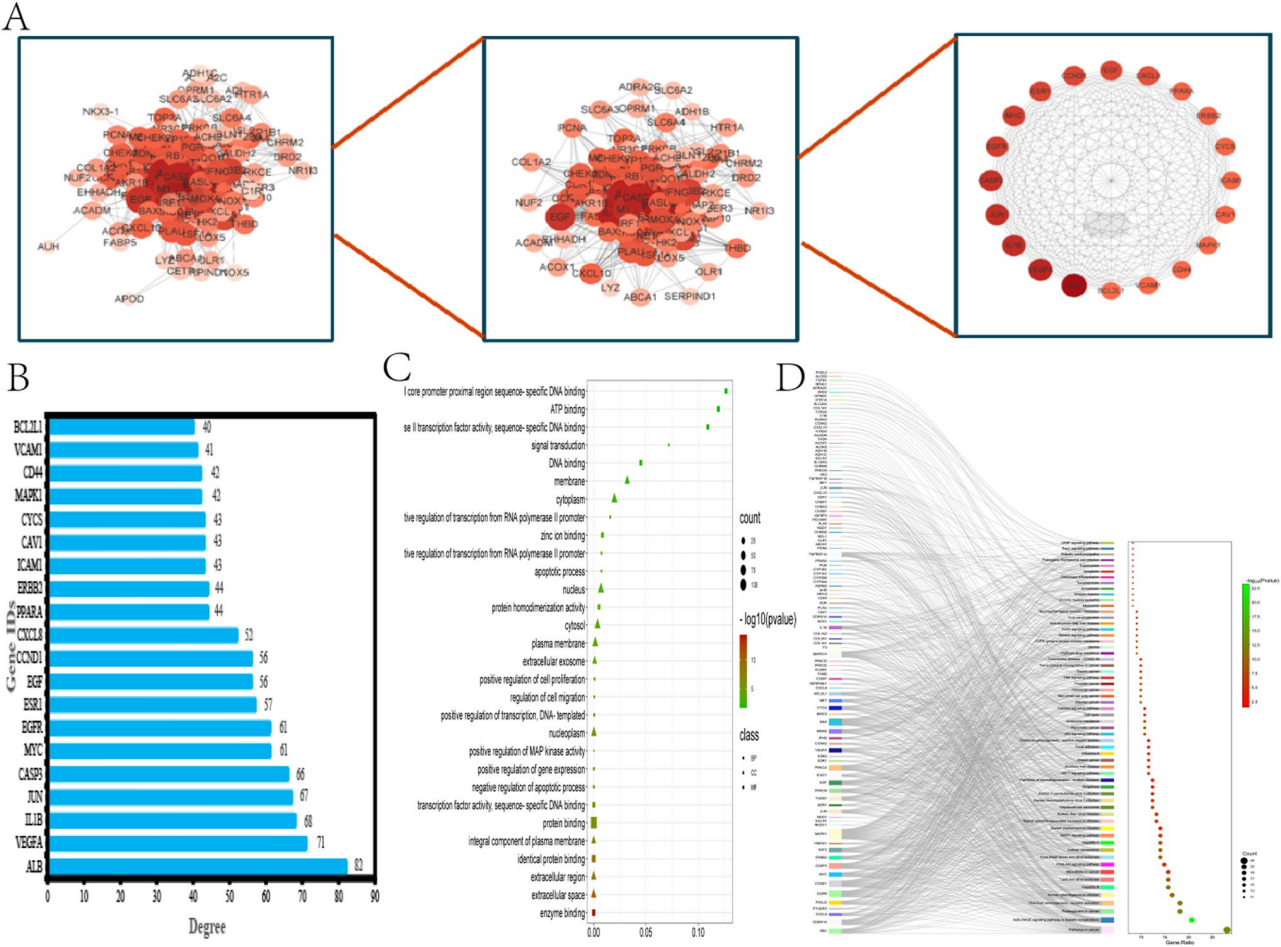
Determination of the hub targets. (**A**) Pivotal targets of the intersection target PPI network, with darker colors indicating higher degree (**B**) The 20 pivotal targets of the PPI network sorted by degree value > 40 (**C**) GO enrichment analysis (**D**) KEGG enrichment analysis of Sankey diagrams

The PPI network results of the intersecting genes showed (Fig. 3A) that the top seven intersecting targets with a degree value > 40 were ALB, VEGFA, IL1B, JUN, CASP3, MYC, and EGFR, which were arranged in order as a bar graph (Fig. 3B). The GO analysis results of the intersecting targets showed that the intersecting targets were mainly associated with negative regulation of apoptotic process, positive regulation of gene expression,positive regulation of transcription, DNA-templated, etc. In cellular components, they are mainly related to cytoplasm, nucleus, cytosol, plasma membrane, etc. In molecular functions, they are mainly related to protein binding, enzyme binding, transcription factor activity, sequence specificity, and so on (Fig. 3C). The results of KEGG sankey map showed that the intersecting targets were mainly related to Pathways in cancer (Fig. 3D).

#### Molecular docking

**Fig. 4:**
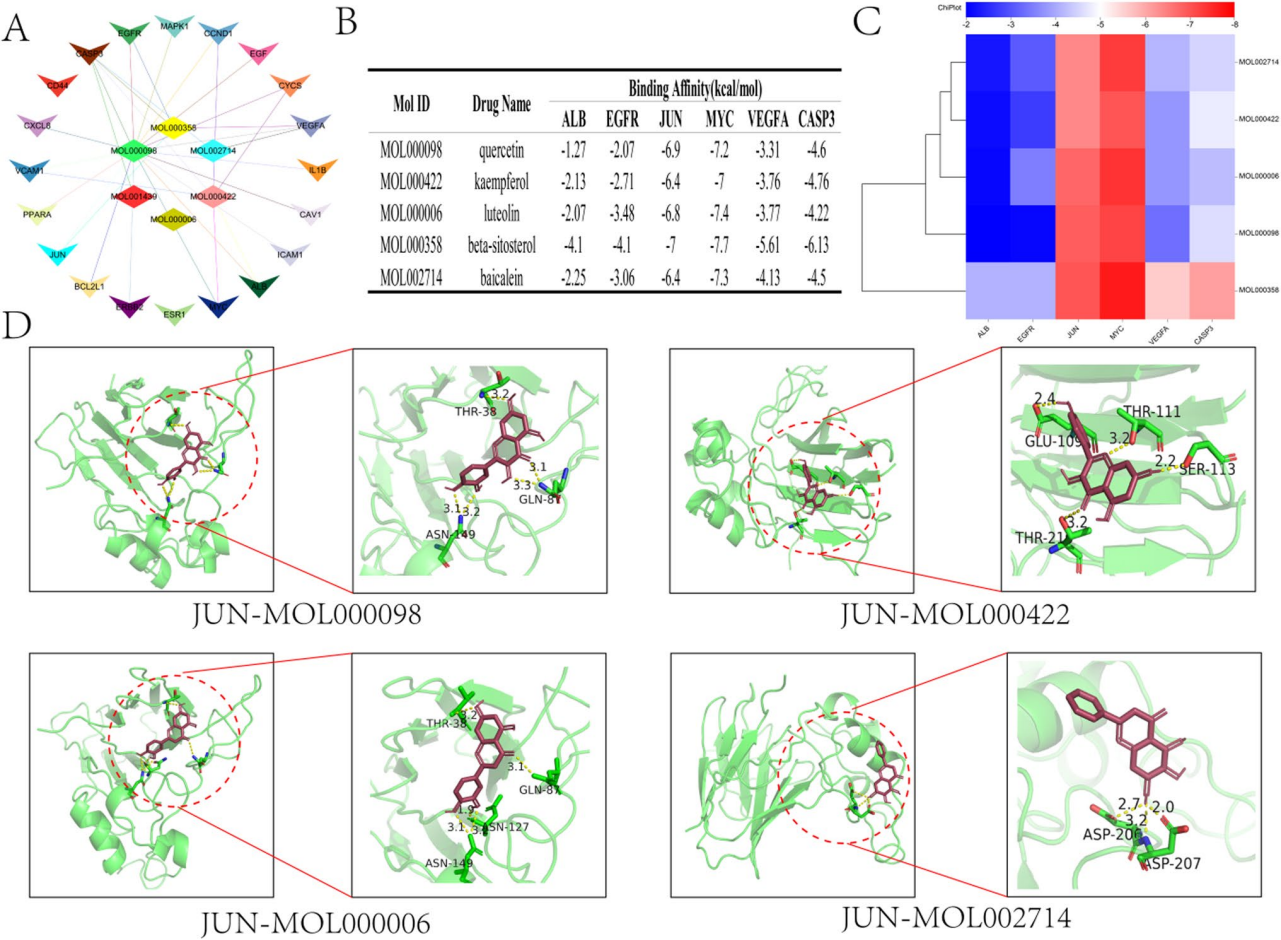
Molecular docking diagram of the core target and the active ingredient. (**A**) PPI network diagram of the top 20 core targets versus the top 5 pharmaceutical active ingredients (**B**) Molecular docking binding energy results (**C**) Heat map of molecular docking binding energy (**D**) Visualization of molecular docking results

The top 20 core targets and the top 5 active ingredient networks ranked by GSY are shown in Fig. 4A. The binding energy of the core target and the key active ingredient is shown in Fig. 4B, and the thermal map of binding energy is shown in Fig. 4C. The results of molecular docking revealed a better binding energy result between the core target and the key active ingredient, which is consistent with our previous prediction. The docking patterns of molecules with higher binding energies are shown in Fig. 4D.

#### Clinical correlation analysis of hub targets

**Fig. 5:**
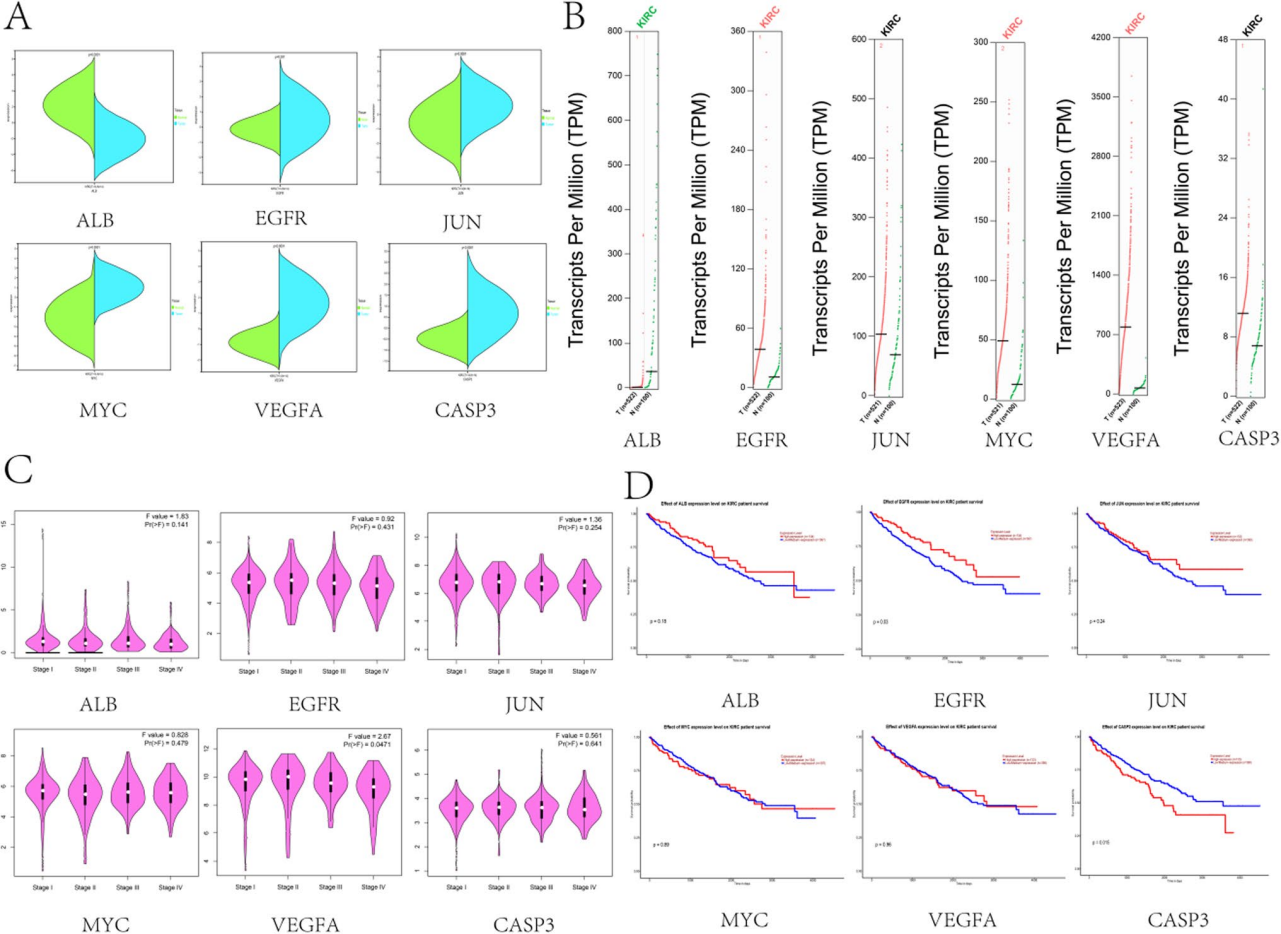
Clinical correlation analysis of core targets. (**A**) Expression of core targets in KIRC tissues and normal kidney tissues (**B**) Copy number changes of core targets, red represents up-regulation and green represents down-regulation (**C**) Significance of core targets in KIRC staging (**D**) Survival analysis of core targets. *p < *0.5*, **p < *0.01*, ***p < *0.001*

The results of hub targets expression in normal kidney tissue and KIRC tissue showed that the expression of ALB in KIRC was lower than that in normal kidney tissue, and the expression of other genes except CASP3 was higher in KIRC than that in normal kidney tissue (Fig. 5A). At the copy number level, ALB was significantly down-regulated and EGFR, MYC, VEGFA were significantly up-regulated (Fig. 5B), which was consistent with the expression results of the KIRC gene chip we obtained. The results of hub targets expression in clinical stage showed that VEGFA had significant statistical difference (Fig. 5C). As shown in Fig. 5D, the correlation between hub targets and overall survival (OS) of patients shows that the expression of EGFR and CASP3 is closely correlated with OS, and the expression level of CASP3 is positively correlated with OS of patients, while the expression level of EGFR is negatively correlated with OS of patients. In other words, the higher the expression of EGFR, the worse the survival ability and the shorter the survival time of patients.

#### Hub targets mutation analysis

**Fig. 6:**
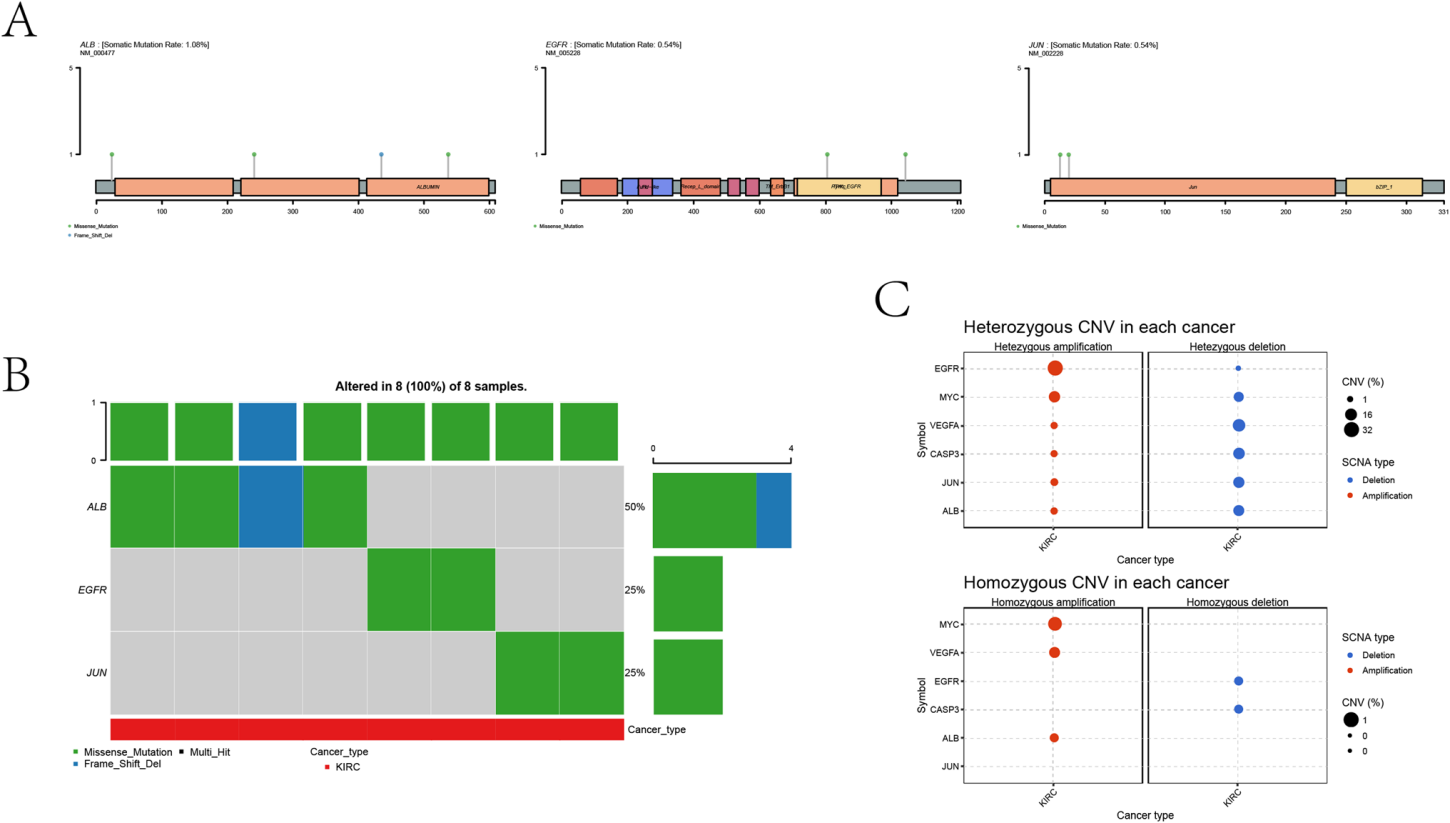
Hub targets mutation analysis. (**A**, **B**, **C**) Mutation types and mutation rates of hub targets

Gene mutation is an important cause of cancer development. The results of mutation types of hub targets showed that missense mutation was dominant in SNV. The somatic mutation rate of ALB was 1.08%, the somatic mutation rate of EGFR and JUN was 0.54% (Fig. 6A), the total mutation rate of ALB was 50%, and that of EGFR and JUN was 25% (Fig. 6B). In CNV, the main types of EGFR mutations are heterozygote amplification, followed by heterozygote deletion and homozygote deletion. The mutation types of MYC were mainly homozygous amplification, followed by heterozygous amplification and heterozygous deletion. The main mutation types of VEGFA, CASP3, JUN and ALB are heterozygote deletion, followed by homozygote, heterozygote amplification and homozygote deletion (Fig. 6C).

#### Methylation level of hub targets and its correlation with immunotherapy

**Fig. 7:**
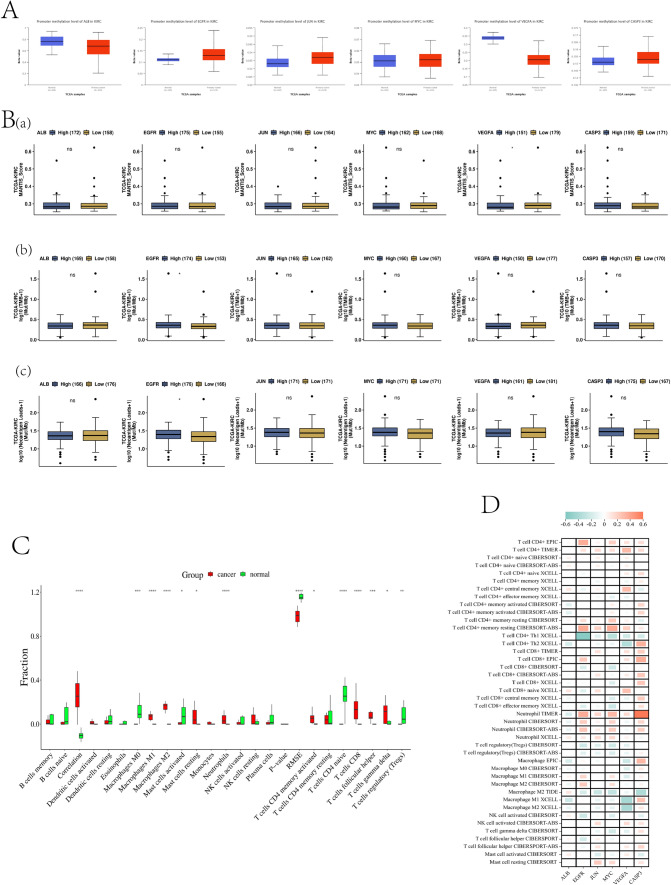
Methylation levels of hub targets and correlation analysis with immunotherapy. (**A**) Methylation level of hub targets (**B**) Immunogenicity analysis of hub targets. (**C**) Immuno-infiltration analysis of KIRC (**D**) Correlation between hub targets and immune cells in KIRC, orange represents positive correlation and green represents negative correlation.*p < *0.5*, **p < *0.01*, ***p < *0.001*

The methylation results showed that in KIRC, the methylation levels of ALB and VEGFA were decreased and the methylation levels of EGFR, JUN, and CASP3 were increased compared with those in normal tissues (Fig. 7A). In the analysis of immunogenic MANTIS score, VEGFA had a higher MANTIS score, which was of great significance in the clinical diagnosis and prognosis of KIRC. In immunogenic TMB analysis, the mutation load of EGFR was higher, indicating that the efficacy of immune checkpoint inhibitors was likely to be better. In the immunogenic NALs analysis, the high expression of EGFR had a higher NALs, indicating that it was beneficial for overall survival and higher immune infiltration (Fig. 7B). The results of immune infiltration showed that the occurrence of KIRC was mainly associated with macrophages (M0, M1, M2), neutrophils, T-cell CD4 naïve, T-cell CD4, and T-cell follicular helper cells (Fig. 7C). Finally, we demonstrated the correlation of hub targets with the major immune cells in KIRC by heatmap, and found that except for ALB and JUN, all targets had significant positive and negative correlation with immune cells in the results of immune infiltration analysis (Fig. 7D).

### Experimental result

#### GSY decreased the viability of 786-O cells and ACHN cells

**Fig. 8:**
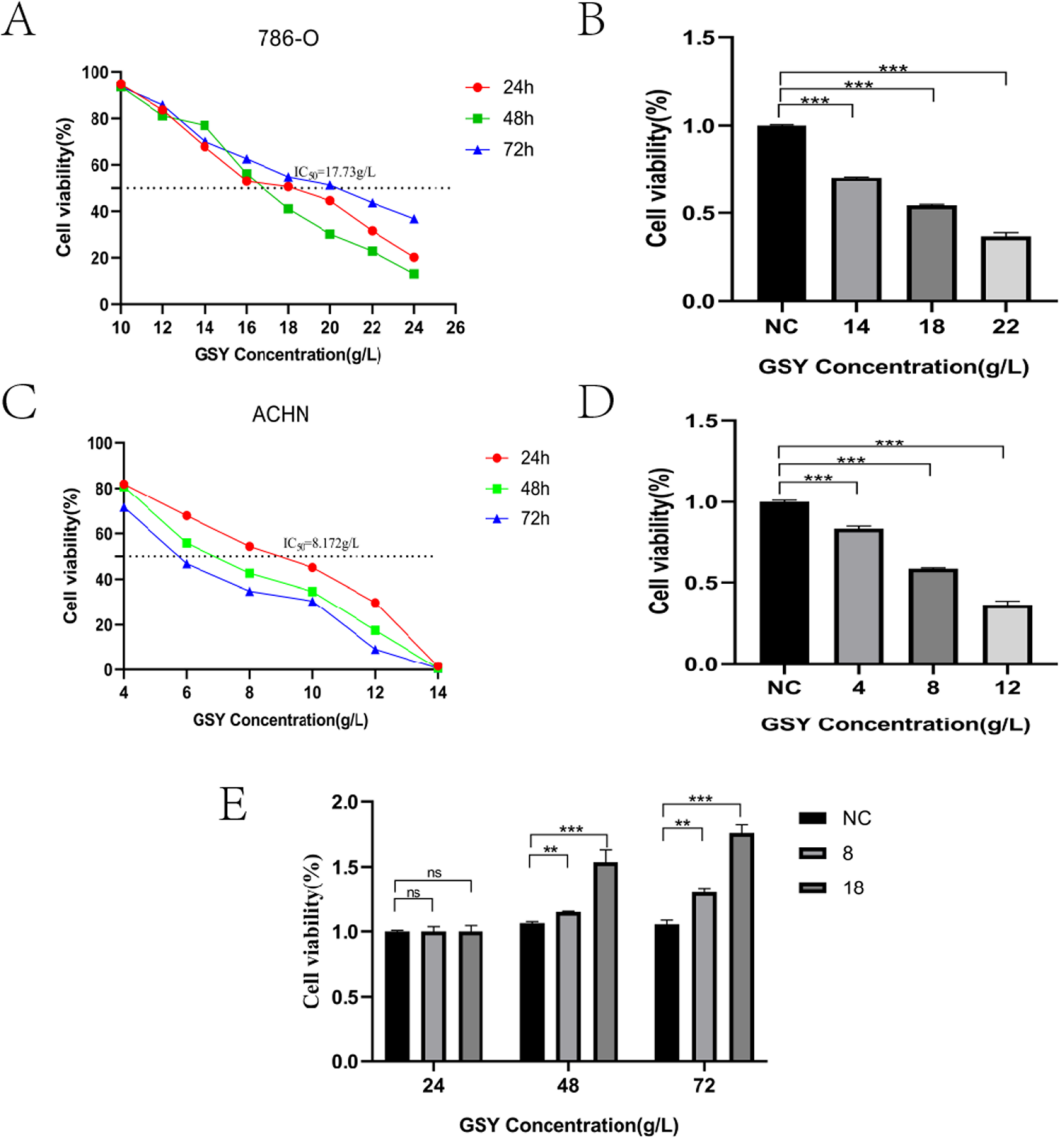
Effect of GSY on the viability of 786O, ACHN and HK-2 cells. (**A**) Effect of GSY on 786-O cell viability at 24, 48 and 72 h after treatment (B) Effect of different concentrations of GSY on 786-O cell viability (**C**) Effect of GSY on ACHN cell viability at 24, 48 and 72 h after treatment (**D**) Effect of different concentrations of GSY on ACHN cell viability (**E**) Effect of GSY on HK-2 cell viability. All experiments were repeated 3 times and data are expressed as mean ± SD, *p < *0.05*, **p < *0.01*, ***p < *0.001*

The results showed that the cell viability of 786-O and ACHN cells decreased significantly after being treated with different concentrations of Gansongyin for 24, 48 and 72 h, respectively (Fig. 8A, C). According to the experimental results, cell proliferation was significantly inhibited after 24 h of GSY intervention, so we chose 24 h as the follow-up time for drug intervention. In order to verify the relationship between the effect of GSY and the change of dose, we selected different concentrations of GSY to treat 786-O and ACHN cells respectively. When the concentration of GSY was 14, 18 and 22 g/L, the inhibition rate of GSY on 786-O cells was close to 25, 50 and 75%, respectively. Therefore, we selected 14, 18 and 22 g/L as the low, medium and high concentration of GSY intervention on 786-O cells (Fig. 8B). When the concentration of GSY was 4, 8 and 12 g/L, the inhibition rate of GSY on ACHN cells was close to 25, 50 and 75%, respectively. Therefore, we selected 4, 8 and 12 g/L as the low, medium and high concentration of GSY intervention on ACHN cells (Fig. 8D). The toxic effect of GSY (8 and 18 g/L) on HK-2 cells was determined, and it was found that it could promote the proliferation of HK-2 cells without drug toxicity. Therefore, follow-up experiments could be continued (Fig. 8E).

#### GSY induced apoptosis in 786-O cells and ACHN cells

**Fig. 9:**
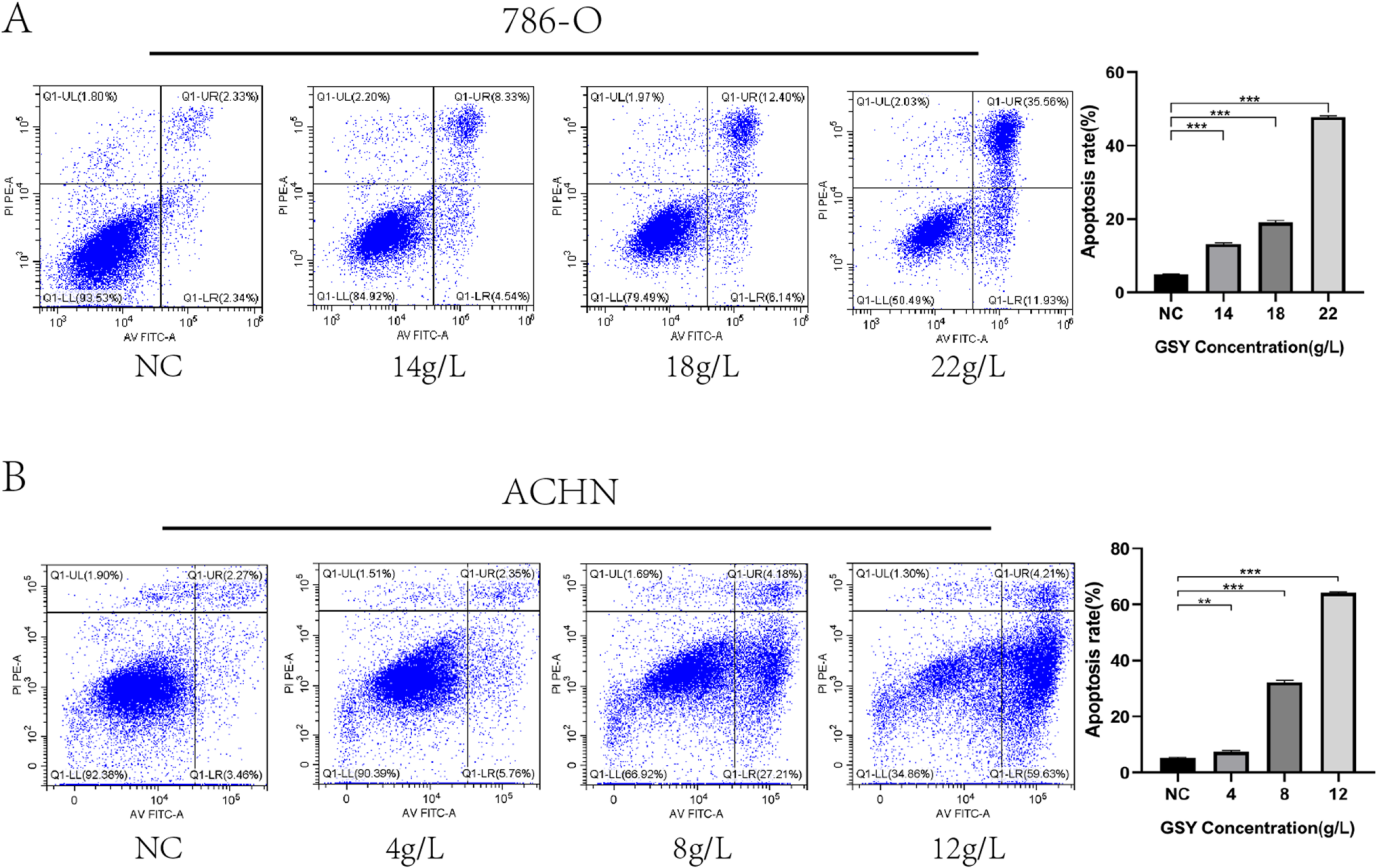
Effect of GSY on apoptosis of 786-O and ACHN cells. (**A**) Effect of GSY on apoptosis of 786-O cells (**B**) Effect of GSY on apoptosis of ACHN cells. All experiments were repeated 3 times and data are expressed as mean ± SD, *p < *0.05*, **p < *0.01*, ***p < *0.001*

The apoptosis results showed that the apoptosis rates of 786-O cells were (13.17 ± 0.63)%, (19.02 ± 1.02)%, (47.77 ± 0.63)%, respectively, after 24 h of intervention of 786-O cells with low (14 g/L), medium (18 g/L), and high (22 g/L) concentrations, and the apoptosis rate of control group was (4.86 ± 0.19)% (Fig. 9A). The apoptosis rates of ACHN cells were (7.42 ± 0.65)%, (32.2 ± 1.27)%, (64.13 ± 0.54)% after 24 h treatment with low concentration (4 g/L), medium concentration (8 g/L) and high concentration (12 g/L), respectively, and the apoptosis rate in the control group was (5.19 ± 0.5)% (Fig. 9B). It was proved that GSY promoted KIRC cell apoptosis.

#### GSY arrested the cell cycle of 786-O and ACHN cells

**Fig. 10:**
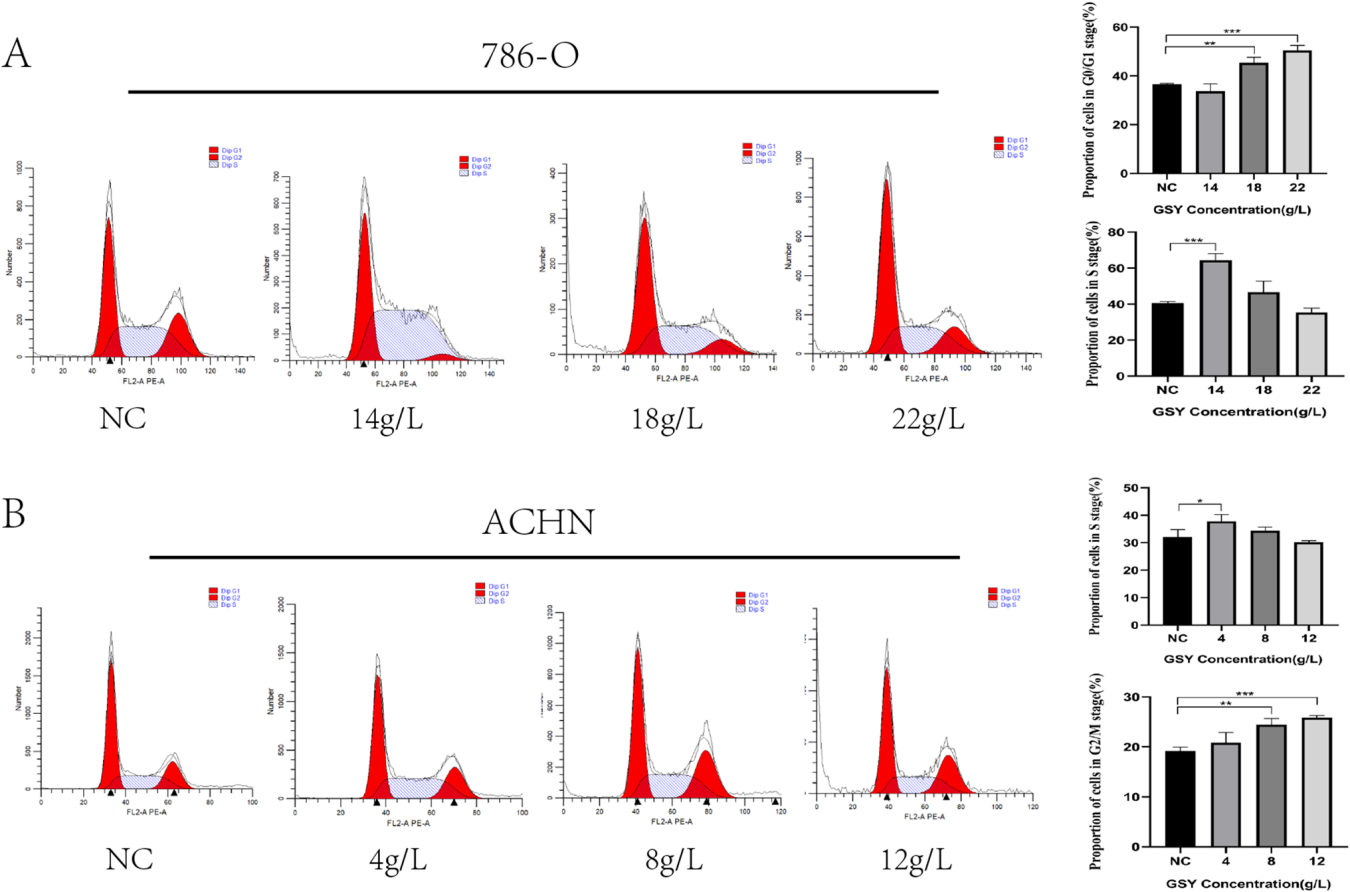
Effect of GSY on cell cycle distribution of 786-O and ACHN. (**A**) Effect of GSY on cell cycle distribution of 786-O (**B**) Effect of GSY on cell cycle distribution of ACHN. All experiments were repeated 3 times and data are expressed as mean ± SD, *p < *0.05*, **p < *0.01*, ***p < *0.001*

The cell cycle results showed that the percentages of 786-O cells in G0/G1 and S phases were significantly changed after 24 h of the action of different concentrations of GSY (14, 18, 22 g/L), and the percentages of G0/G1 phase were (45.37 ± 2.29)% and (50.45 ± 2.02)%, respectively, which were all higher as compared with the control group (36.64 ± 0.31)%. The percentages of S phase was (64.35 ± 3.66)%, which was higher than that of the control group (40.62 ± 0.78)% (Fig. 10A). After 24 h of action of different concentrations of GSY (4, 8 and 12 g/L), the percentages of ACHN cells in S and G2/M phases changed significantly, and the percentage of S phase was (37.82 ± 2.46)%, which was more than that of the control group (32.08 ± 2.68)%. The percentages of G2/M phase were (24.47 ± 1.23)% and (25.86 ± 0.44)%, which were both higher than the control group (19.16 ± 0.78)% (Fig. 10B).

#### GSY inhibited the colony formation ability of 786-O cells and ACHN cells

**Fig. 11:**
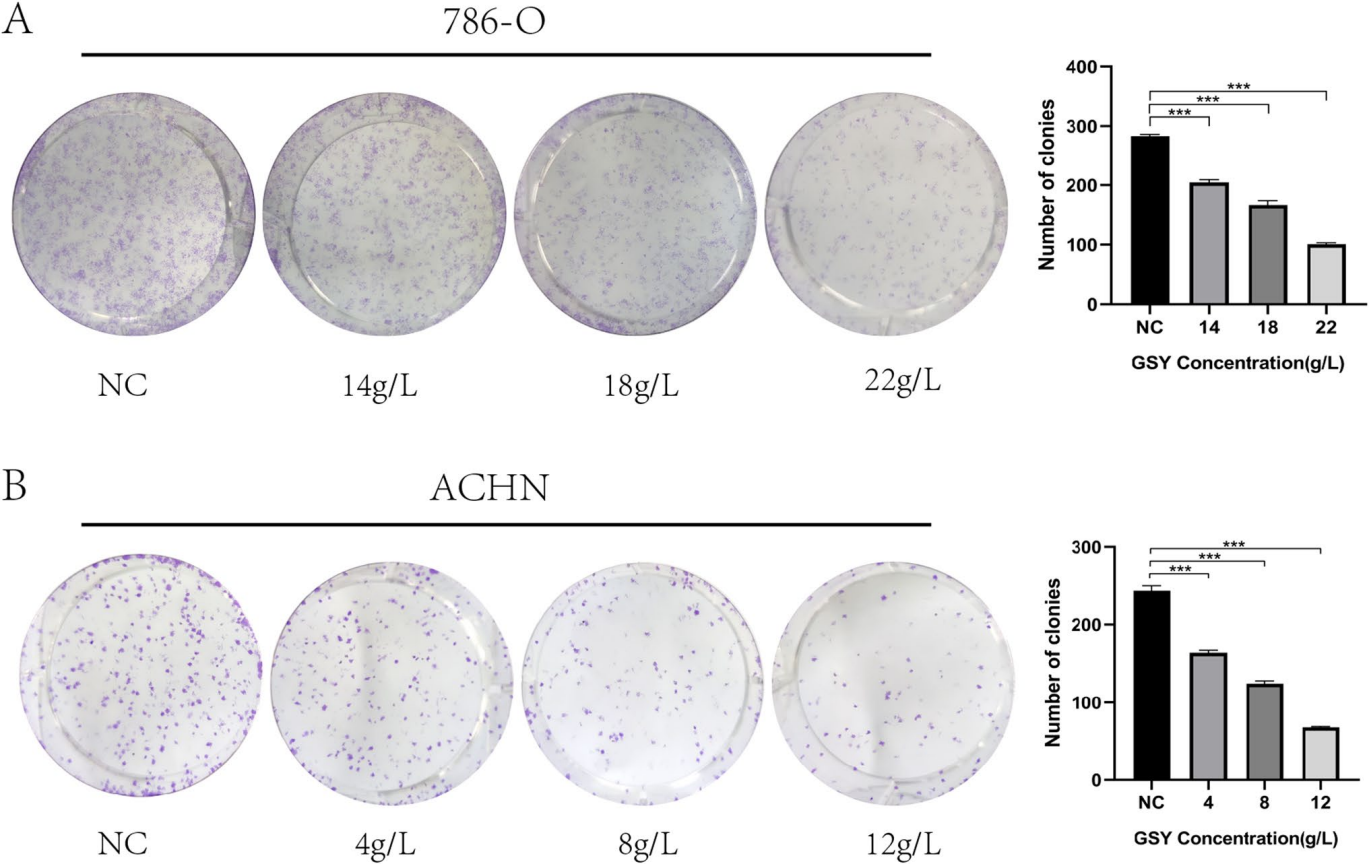
Effect of GSY on clonal formation ability of 786-O and ACHN cells. (**A**) Effect of GSY on clonal formation ability of 786-O cells (**B**) Effect of GSY on clonal formation ability of ACHN cells. All experiments were repeated 3 times and data are expressed as mean ± SD, *p < *0.05*, **p < *0.01*, ***p < *0.001*

The results of plate cloning formation experiment showed that compared with the control group, the number of cell clones formed after GSY treated 786-O and ACHN cells respectively decreased significantly, and the inhibition effect was more obvious with the increase of concentration (Fig. 11A, B).

#### GSY inhibited the expression of hub targets

**Fig. 12:**
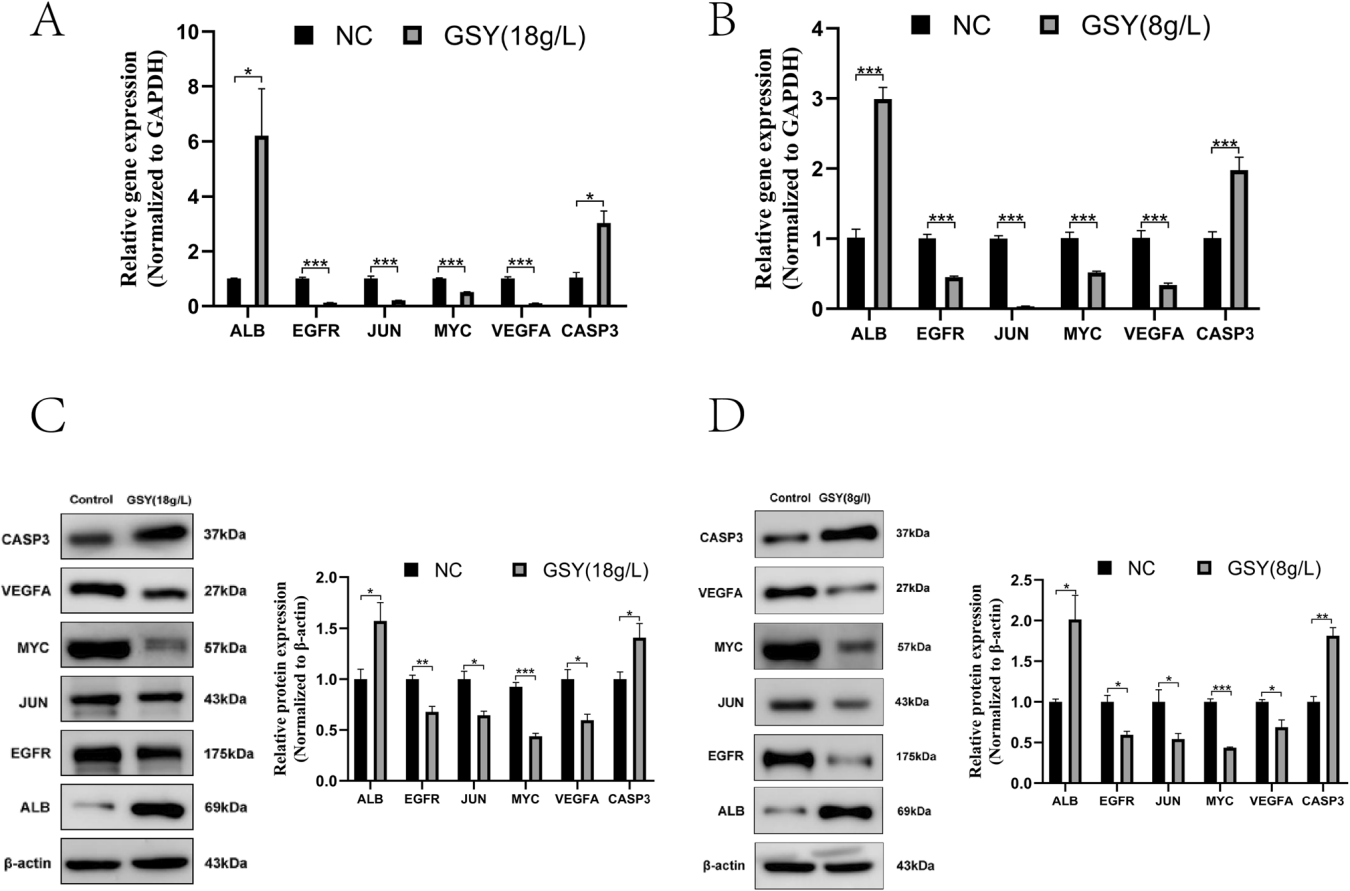
GSY inhibited the expression of hub targets. (**A**) Effect of GSY on core target mRNA expression in 786-O cells (**B**) Effect of GSY on core target mRNA expression in ACHN cells (**C**) Effect of GSY on core target protein expression in 786-O cells (**D**) Effect of GSY on core target protein expression in ACHN cells. All experiments were repeated 3 times and data are expressed as mean ± SD, *p < *0.05*, **p < *0.01*, ***p < *0.001*

According to the results of network pharmacology and bioinformatics analysis, we found that GSY may achieve therapeutic effects on KIRC by regulating the expression of core targets of ALB, VEGFA, JUN, CASP3, MYC and EGFR. The results of qRT-PCR showed that after GSY intervention, the expression levels of *ALB* and *CASP3* increased, while the expression levels of *EGFR*, *JUN*, *MYC* and *VEGFA* decreased (Fig. 12A, B). Western blot results showed that after GSY intervention, the level of protein expression of ALB, CASP3 was increased, and the level of protein expression of EGFR, JUN, MYC, VEGFA was decreased (Fig. 12C, D).

## Materials and methods

### Network pharmacology and bioinformatics analysis

#### Prediction of GSY and KIRC targets

The active ingredients and targets of GSY were obtained from TCMSP (https://old.tcmsp-e.com/tcmsp.php), and target names were converted to generic gene names using the Uniprot (https://www.uniprot.org/) database. The active ingredients of various TCM in GSY were drawn into a rose diagram. The PPI network of GSY components and corresponding targets was constructed by Cytoscape 3.8.2 software. “KIRC” and “ Renal clear cell carcinoma” were used as keywords to search for KIRC related targets in the GEO (https://www.ncbi.nlm.nih.gov/) database, and GSE66270 dataset was selected as the research object. The data contained 14 pairs of KIRC and adjacent tissues. The differentially expressed genes (DEGs) between KIRC group and adjacent group were screened out (LogFC ≥|1| and p-value < 0.05 as screening criteria), and the volcano map of differentially expressed genes (DEGs) was drawn by Prism8 software. The up-and down-regulated genes were intersected with the targets of GSY, respectively. In the GeneCards (https://www.genecards.org/) database with relevance score > 10 for the conditions to obtain KIRC targets. The GSY targets after duplication removal were summarized and the venn diagram of GSY-KIRC-DEGs was constructed on the W bioinformatics website (http://www.bioinformatics.com.cn/) to obtain the intersection targets. The KIRC microarray data were used to draw a heat map of the intersection targets.

#### Determination of the hub targets

The PPI network files of intersection targets were downloaded from STRING (https://cn.string-db.org/) database, and then the PPI network diagram of intersection targets was constructed on Cytoscape3.8.2 software. Finally, Prism8 software was used to show the top 20 intersection targets with degree value greater than 40. The intersection targets were annotated in the DAVID (https://david.ncifcrf.gov/) database, and the annotated results were analyzed by GO and KEGG enrichment analysis using W bioinformatics and Sangerbox 3.0 (http://sangerbox.com/home.html).

#### Molecular docking

The PPI network of the top 20 core targets with the top 5 active ingredients ranked by GSY was constructed in Cytoscape_v3.8.2 software, then the top 5 3D structures were obtained in PubChem (https://pubchem.ncbi.nlm.nih.gov/) database, and finally the crystal structures were selected as receptors in the PDB (https://www.rcsb.org/) database. PYMOL (https://pymol.en.softonic.com/) software was used to remove water molecules and ligands from the crystals, and then AutoDock Tools (http://autodock.scripps.edu/) software and AutoDock Vina (https://vina.scripps.edu/) were used to perform molecular docking. The docking energy results were visualized, and the protein residue names and hydrogen bond sizes were labeled.

#### Clinical correlation analysis of hub targets

Differential expression of hub targets in KIRC and normal tissues was analyzed in the Sangerbox 3.0 database. Gene Expression Profile, Pathological Stage, and Survival Plots of hub targets were utilized in the GEPIA database (http://gepia.cancer-pku.cn/).

#### Hub targets mutation analysis

The somatic mutation rate of the hub targets and the mutation sites and types of single nucleotide mutation (SNV) and copy number mutation (CNV) were analyzed by using the “Mutation” module in the GSCA (http://bioinfo.life.hust.edu.cn/) database.

#### Methylation level of hub targets and its correlation with immunotherapy

Through the “TCGA” module in the UALCAN (https://ualcan.path.uab.edu/) website, the hub targets methylation expression box map was drawn. Immunogenicity analysis was performed in the “Immunogenicity” module in the CAMOIP (http://www.camoip.net/) database and visualized using a box plot. The effect of immune cells on KIRC was obtained through the CIBERSORT (Thttps://cibersortx.stanford.edu/) database, and the downloaded data was sorted out and imported into W bioinformatics for visual analysis. The correlation between hub targets and immune cells in KIRC was analyzed on the TIMER 2.0 (http://timer.cistrome.org/) website, and visualized in the ChiPlot (https://www.chiplot.online/) database. The results were finally presented in the form of heat maps.

### Experimental verification

#### Experimental materials and reagents

The experimental materials and reagents are shown in Table 1, and the antibodies required for the experiment are shown in Table 2.

**Table 1:** Materials and reagents

Materials and reagents	Manufacturer	Item number/lot number
Human renal clear cell adenocarcinoma cell 786-O	Shanghai Fuheng Biotechnology Co., LTD	FH0229
Human renal cell adenocarcinoma cell ACHN	Shanghai Fuheng Biotechnology Co., LTD	FH0549
Human renal cortex proximal convoluted tubule epithelial cells HK-2	Wuhan Punosai Life Technology Co., LTD	CL-0109
GSY formula granules	Ningxia Medical University Affiliated Hospital of Traditional Chinese Medicine	
Fetal bovine serum	GEMINI	900108
RPMI-1640 medium	Gibco	1187517
MEM medium	Wuhan Punosai Life Technology Co., LTD	PM150411B
cck-8 kit	Dojindo	CK04
Cell cycle test kit	Jiangsu Kaiji Biotechnology Co., LTD	KGA512
Cell apoptosis detection kit	Jiangsu Kaiji Biotechnology Co., LTD	KGA107
Crystal violet	Beijing Boaotoda Co., LTD	548-62-9
Trizol	Tianjin Biochemical Technology Co., LTD	DP419
Reverse transcription reagent	Dalian Takara Company	RR047A
Real-time fluorescent quantitative reagent	Dalian Takara Company	FP205
Total protein extraction kit	Jiangsu Kaiji Biotechnology Co., LTD	KGP250

**Table 2:** Antibody name and origin

Antibody	Manufacturer	Article number
ALB	Affinity	DF6396
CASP3		AF6311
VEGFA		DF7470
EGFR	Chengdu Zhengneng Biotechnology Co., LTD	R22778
JUN		380397
MYC		R22809

#### Cell culture

786-O cells were cultured with 90% RPMI-1640 + 10% FBS + 1% penicillin–streptomycin mixed antibiotics. ACHN cells were cultured with 90% MEM medium + 10% FBS + 1% penicillin–streptomycin mixed antibiotics. Finally, the cells were cultured in a humidified incubator of 5% CO2 at 37 ℃ and cultured every 2–3 days.

#### Determined by cell viability assay

786-O cells (6000 cells/well) and ACHN cells (8000 cells/well) were homogeneously inoculated in 96-well plates (each well/100 uL medium). The experiment was separated into three groups: blank group, check group and drug group. GSY (12, 14, 16, 18, 20, 22, 24) g/L and (4, 6, 8, 10, 12, 14) g/L solutions at different concentrations were used to act on 786-O cells and ACHN cells for 24, 48, and 72 h, respectively. CCK-8 solution was added at well/10 μl and incubated for 4 h. The toxic effect on HK-2 cells (1^*^10^4^ cells/well) was determined by GSY afterward.The optical density (OD) of each group of cells was determined by an enzyme labeling instrument. Cell viability = [(absorbance of drug group—absorbance of blank group)]/[(absorbance of control group—absorbance of blank group)] × 100%.

#### Cell apoptosis detection

786-O (1.2^*^10^5^ cells/well) and ACHN (1.6^*^10^5^ cells/well) cells were placed in 6-well plates for the experiment, and when the cell adhesion growth was 70–80%, GSY solution (0, 14, 18 and 22 g/L)/(0, 4, 8 and 12 g/L) was added for 24 h, respectively. Finally, the supernatant and cells were collected, and the cells were washed with PBS twice. Reagents were added according to the apoptosis kit, and the reaction was kept away from light at room temperature for 5–15 min. The analysis was performed by flow cytometry within 1 h.

#### Cell cycle analysis

The cells were placed on a 6-well plate for 24 h and then treated synchronously for 8 h. Finally, different concentrations of drug-containing medium were added for intervention. After 24 h, the supernatant was discarded and cell precipitates were collected. After 2 times of cleaning with PBS, 1 ml of pre-cooled 70% ethanol was added to fix the cells and placed at 4 ℃ overnight. The next day, the ethanol was centrifuged and washed twice with PBS. Then the reagent was added according to the cycle kit, and the reaction was kept away from light at room temperature for 30–60 min. Cell cycle percentage was measured by flow cytometry.

#### Plate clone experiment

The cells were inoculated into 6-well plates with 500 cells per well. After the cells were attached to the wall for 24 h, drug-containing medium was added. After 24 h of intervention, the medium was changed for 10–14 days, and the medium was changed every 3 days. After clonal formation and termination culture, 4% paraformaldehyde was added and fixed at 4 ℃ for 30 min, and crystal violet solution was added. After dyeing at room temperature for 15 min, wash the dyeing solution with PBS.

#### qRT-PCR experiment

RNA was extracted from each group by Trizol, and RNA concentration and purity were detected by Nanodrop 2000C. RNA was reversed-transcribed into cDNA using reverse transcriptional kit, and then amplified upstream by real-time fluorescence quantification kit. *GAPDH* was used to detect the expression level of hub genes. The primers are shown in Table 3. The relative expression levels of hub genes in 786-O and ACHN cells were analyzed by 2^−△△Ct^.

**Table 3:** Primer sequences

Gene Name	primer sequence (5ʹ → 3ʹ)
*CASP3*	Forward 5ʹ-TGGAAGCGAATCAATGGACTCTGG-3ʹ
	Reverse 5ʹ-AGACCGAGATGTCATTCCAGTGC-3ʹ
*VEGFA*	Forward 5ʹ-GCCTTGCTGCTCTACCTCCAC-3ʹ
	Reverse 5ʹ-GATGATTCTGCCCTCCTCCTTCTG-3ʹ
*MYC*	Forward 5ʹ-GTCTGGATCACCTTCTGCTGGAG-3ʹ
	Reverse 5ʹ-GCTGCGTAGTTGTGCTGATGTG-3ʹ
*JUN*	Forward 5ʹ-AGAACTCGGACCTCCTCACCTC-3ʹ
	Reverse 5ʹ-ATGTGCCCGTTGCTGGACTG-3ʹ
*EGFR*	Forward 5ʹ-GTCTTGAAGGCTGTCCAACGAATG-3ʹ
	Reverse 5ʹ-CACCACCAGCAGCAAGAGGAG-3ʹ
*ALB*	Forward 5ʹ-GTTTCGTCGAGATGCACACAAGAG-3ʹ
	Reverse 5ʹ-TGAGCAAAGGCAATCAACACCAAG-3ʹ
*GAPDH*	Forward 5ʹ-CAGGAGGCATTGCTGATGAT-3ʹ
	Reverse 5ʹ-GAAGGCTGGGGCTCATTT-3ʹ

#### Western blot analysis

The cells after drug treatment were collected and the total protein was extracted by adding lysate according to the instructions of the kit. The protein content was determined with BCA protein quantitative kit, and the protein was denatured at 100 ℃ for 10 min. After SDS-PAGE, the target protein was transferred to PVDF membrane, and 5% skim milk powder was closed for 2 h. After washing with TBST for 3 times, add primary antibody and incubate at 4 °C overnight. The PVDF membrane was cleaned the next day and incubated at room temperature for 1.5 h after adding the second antibody. Finally, the protein was detected by ECL luminescent solution and analyzed by Image J.

#### Statistical method

All experiments in this chapter are 3 independent repeated experiments, and the data are the average of 3 independent repeated experiments. GraphPad prism 8.0 software was used to statistically analyze the experimental data, and the data used were expressed as mean ± standard deviation. Comparisons between two groups were made using t-test, and comparisons between multiple groups were made using one-way ANOVA, with *p* < *0.05* being considered statistically significant.

## Discussion

Due to the high incidence and rapid progression of KIRC, many patients are deprived of the option of cure or surgical resection and some of them are diagnosed at a late stage due to the absence of obvious symptoms [15]. Although surgical resection improves patient survival and other adjuvant therapies improve prognosis, KIRC patients usually have a poorer prognosis and more side effects [16], so the search for drugs with good anticancer effects and reduced side effects has become a new research goal and hot spot. Based on the therapeutic characteristics of Chinese medicine as “multi-component, multi-target, and multi-pathway”, GSY has also been extensively used in the research and treatment of renal diseases, but the related research on KIRC has not yet been reported. Therefore, this paper aims to reveal the mechanism of action of GSY in the treatment of KIRC through network pharmacology, bioinformatics and experimental validation, so as to make a modest contribution to the treatment of KIRC by TCM.

Network pharmacology results suggest that the key active ingredients for the treatment of KIRC mainly include quercetin, kaempferol, lignans and baicalein. Quercetin is a natural flavonoid, which is widely distributed in plants, foods and Chinese herbal medicines. Recent years, quercetin can effectively inhibit the proliferation of renal carcinoma 786–0 cells, and its anti-tumor effect is mainly achieved by inducing apoptosis of tumor cells [17]. Kaempferol is a flavonoid with anti-tumor and antioxidant damage functions, which not only participates in a wide range of bioprocesses, such as cell proliferation [18], apoptosis [19], cell cycle [19] and autophagy [20], but also inhibits proliferation of human renal carcinoma 786-O cells, reduces the expression of PCNA and VCAM-1, and induces 786-O cells to block in the S-phase and G2/M-phase [21]. Luteolin has anti-tumor, antibacterial, antioxidant and other pharmacological effects. It can inhibit tumor growth by inhibiting tumor cell proliferation, invasion and metastasis, blocking tumor cell cycle, promoting tumor cell apoptosis and regulating tumor cell reactive oxygen species (ROS) level, thus inhibiting the occurrence and development of various tumors in the body, and has good anti-tumor effects [22]. Baicalein is a kind of naturally occurring flavonoid extracted from scutellaria baicalensis.It has significant anti-tumor effects such as inhibiting tumor cell activity and inhibiting tumor cell proliferation in a variety of malignant tumors [23]. In this study, PPI networks were constructed for the intersection targets of GSY's active ingredients and KIRC through network pharmacology and bioinformatics, and it was found that ALB, CASP3, MYC, EGFR, JUN, VEGFA, etc., were the core anti-KIRC targets. Therefore, we presumed that GSY could achieve the purpose of treating KIRC by regulating the expression of the above core targets.

To further reveal the mechanism of action of GSY for the therapy of KIRC, we validated the core targets using an in vitro cellular assay approach. We conducted CCK-8 experiments with 786-O cells and ACHN cells, and the results showed that the viability of 786-O and ACHN cells decreased after 24 h of GSY intervention, indicating that GSY had a significant inhibitory effect on 786-O and ACHN cells in a dose—and time-dependent manner. Apoptosis is a kind of death mechanism which is closely regulated by many apoptosis genes [24]. The results showed that the apoptosis rate of 786-O cells and ACHN cells increased with the increase of GSY drug concentration compared with the control group, indicating that GSY significantly induced apoptosis in 786-O and ACHN cells in a dose-dependent manner. Cell cycle alterations play an important role in tumor development [25]. The experimental results showed that with the increase of GSY concentration, the number of G0/G1 and S phase cells in 786-O cells increased, and the number of S and G2/M phase cells in ACHN cells increased. These results indicated that GSY could block 786-O cell cycle in G0/G1 and S phases and ACHN cell cycle in S and G2/M phases, thereby inhibiting cell proliferation. The results of plate clone formation experiment showed that the number of cells in GSY group was significantly reduced compared with the control group, indicating that GSY could significantly inhibit the colony-forming ability of 786-O and ACHN cells and thus inhibit cell proliferation. Albumin (ALB) is one of the most important proteins in human plasma and is a known nutritional status marker. It has been evaluated as an independent predictor of several cancers, including renal cell carcinoma [26]. Caspase-3 (CASP3) is the most important terminal cleavage enzyme in the process of apoptosis. WOO et al. [27] found that curcumin treatment of Caki cells can lead to CASP3 activation and induce apoptosis. JUN, also known as c-Jun on Gene card, is shown to exert an influential force in cytokine signaling, growth, polarization, or in the apoptotic process. The MYC oncogene (also known as c-Myc) is part of a superfamily of genes whose products are among the most commonly activated in human cancers [28]. Epidermal growth factor receptor (EGFR) is a member of the ErbB family of receptor tyrosine kinases (RTKs), which play key functions in epithelial cell physiology [29]. Due to its frequent mutation and/or overexpression in different types of human cancers, it is not only a target for a wide range of cancer therapies currently employed in clinical practice [30], but is becoming more commonly recognized as a biomarker of drug resistance in tumors [31]. Vascular endothelial growth factor A (VEGFA), a key angiogenic factor, is an important tumor-specific factor in RCC patients and plays a crucial role in tumor angiogenesis and progression [32]. Preclinical studies have reported that VEGFA is overexpressed in patients with RCC [33, 34], and reducing its expression may be a new means of clinical treatment for renal cell carcinoma. In this paper, the effects of GSY on core genes and proteins in 786-O cells and ACHN cells were detected by qRT-PCR and Western blot experiments. The results showed that GSY up-regulated the expression of *ALB* and *CASP3* genes, and down-regulated the expression of *EGFR*, *JUN*, *MYC* and *VEGFA* genes. The expression levels of ALB and CASP3 were up-regulated, and the expression levels of EGFR, JUN, MYC and VEGFA were down-regulated, suggesting that GSY inhibited the proliferation of 786-O cells and ACHN cells by regulating the expression of hub targets.

In conclusion, GSY can inhibit the proliferation of KIRC cells by regulating the expression of hub targets, promoting KIRC cell apoptosis and cell cycle arrest, and reducing the ability of cell clonal formation. However, due to time and financial constraints, we have limited our study to in vitro cellular experiments at this time, and more experiments are needed to support our future studies. Therefore, we will keep exploring the link between GSY and KIRC in our future studies. Firstly, we will investigate the therapeutic effect of GSY on KIRC through in vivo animal experiments. Secondly, we will explore the role of GSY in combination with chemotherapeutic drugs to lower the sensitivity of chemotherapeutic drugs. Finally, the mechanism of GSY in treating KIRC will be further investigated through Lentiviral transfection and co-IP.

## Conclusions

In this study, we explored the molecular mechanism of GSY for the treatment of KIRC through network pharmacology, bioinformatics and experimental validation. The results showed that GSY could inhibit the proliferation of KIRC cells by regulating the expression of hub targets to induce apoptosis and cell cycle arrest and reduce colony forming ability. Our results not only confirmed the reliability and authenticity of the network pharmacology and bioinformatics analysis, but also will form a scientifically sound basis for further research.

## Data Availability

Data on the active ingredients and targets of action of GSY were obtained from TCMSP (https://old.tcmsp-e.com/tcmsp.php). Data on renal clear cell carcinoma were obtained from GEO (https://www.ncbi.nlm.nih.gov/geo/query/acc.cgi?acc=GSE66270) and Genecards (https://genecards.weizmann.ac.il/v3/index.php?path=/Search/keyword/clear%20cell%20carcinoma%20of%20kidney).

## Abbreviations

GSY: Gan-song Yin
KIRC: Renal clear cell carcinoma
TCM: Traditional Chinese Medicine
TCMSP: Traditional Chinese Medicine Systems Pharmacology Database and Analysis Platform
PPI: Protein–protein interaction
GO: Gene Ontology
KEGG: Kyoto Encyclopedia of Genes and Genomes
CCK-8: Cell Counting Kit-8 kit
qRT-PCR: Quantitative real-time polymerase chain reaction
SNV: Single nucleotide mutation
CNV: Copy number mutation
